# The Role of Calcium, Lipid Membranes and Islet Amyloid Polypeptide in the Onset of Type 2 Diabetes: Innocent Bystanders or Partners in a Crime?

**DOI:** 10.3389/fendo.2014.00216

**Published:** 2014-12-17

**Authors:** Danilo Milardi, Michele F. M. Sciacca, Loredana Randazzo, Antonino Raudino, Carmelo La Rosa

**Affiliations:** ^1^Istituto CNR di Biostrutture e Bioimmagini-Sezione di Catania, Catania, Italy; ^2^Dipartimento di Scienze Chimiche Viale Andrea Doria 6, Università Degli Studi di Catania, Catania, Italy

**Keywords:** type II diabetes mellitus, amyloid, membranes, calcium, obesity, free fatty acids, genetic predisposition

## Introduction

Type II diabetes mellitus (T2DM) is one of the most disabling age-related pathologies in developed countries (1). Its incidence is constantly growing: the number of people affected by T2DM worldwide grew from 28 million cases in 1985 to around 168 million in 2001, with an estimate of 350 million cases in 2030 (2).

Despite the intense research efforts focusing on T2DM in the last 20 years, the cause of the underlying pathology is still unclear. Islet Amyloid Polypeptide (IAPP) is an amyloidogenic protein, member of the calcitonin family (3), which is known to be involved in the mechanisms lying at the root of T2DM pathogenesis (4, 5). Indeed, although the correlation between islet β-cell death and amyloid fibril formation is still a matter of debate, recent studies, performed on transgenic mice, show that IAPP aggregation can mediate β-cell failure in T2DM (6). Indeed, despite human and rat variants of IAPP differing by only six amino acids, the two variants behave in a completely different way. In particular, rat IAPP (rIAPP) is unable to form amyloid aggregates under normal conditions (7) and rats do not develop T2DM in analogous conditions. Human IAPP (hIAPP) amyloid aggregates are detectable in pancreas’ extracellular space in 90% of patients (4), while only 10% of patients do not have detectable amyloid deposits.

We propose that T2DM etiology might be clarified by studying the close relationships between three apparently independent issues: (i) amyloid toxicity; (ii) genetic factors; and (iii) environmental risk factors related to lifestyle and diet.

## Mechanisms of IAPP-Induced Membrane Disruption

The mechanisms by which amyloid intermediates (Aβ peptide, hIAPP, and other amyloid-forming proteins) cause cytotoxicity and disease remains unsolved, although some recent studies (8) suggest that the water/membrane interface is critical for influencing amyloid aggregation. Of note, mature amyloid fibrils are presumed to be inert while small oligomeric intermediates are suggested to penetrate the cell membrane (9). It has been hypothesized that amyloid-forming peptides may cause membrane damage by: (a) changing the bilayer fluidity, (b) generating protein-stabilized pores (poration), (c) laying on one leaflet of the membrane (carpeting), or (d) removing lipid components from the bilayer by detergent-like mechanisms (10). It has also been shown that the formation of amyloid fibers occurs independently of membrane leakage and that membrane composition and the presence of ions may have a major influence on amyloid-mediated membrane damage (11).

In recent years, several studies have focused on the interpretation of molecular mechanisms underlying T2DM. In particular, researchers investigated the interaction between hIAPP and phospholipid membranes, a process that seems to be correlated to the loss of pancreatic β-cells (12–15). The most recent results suggest that hIAPP-induced membrane disruption could be described as a two independent step process. The first step is correlated with pore formation occurring after the insertion of monomeric or oligomeric species inside the membrane hydrophobic core. The insertion of a protein into a membrane interferes with lipid packing and, at high concentration, it might result in a local bending deformation. To minimize the energetically unfavorable deformation, proteins tend to cluster (16). The second step of membrane disruption is correlated with the growth of fibers onto the membrane surface, which causes complete membrane dissolution through a detergent-like mechanism (13, 17).

Since the two steps are independent processes, they might be separately targeted by novel therapeutic approaches. The membrane lipid composition has been shown to be a critical point for the mechanism of membrane disruption mediated by hIAPP. The presence of phosphatidylethanolamine headgroup lipids, for example, can modulate the entity of the first and second step of membrane disruption. Also, the presence of cholesterol could finely regulate membrane dissolution by both inhibiting the affinity of hIAPP for the membrane as well as its insertion in the bilayer. Unfortunately, to date there are a lack of studies that have systematically investigated the role played by membrane composition (i.e., length of hydrophobic tails, percentage of cholesterol, presence of different lipid headgroups) on hIAPP/membrane interactions.

Therefore, the membrane disruption mechanism can be regulated by several factors, such as metal ions balance, increased hIAPP secretion, the presence of free fatty acid, etc. which could arise from genetic predisposition, lifestyle, and diet.

## Genetic Risk Factors in the Development of T2DM: Insulin and Calcium Receptors

Despite enormous efforts, the precise identification of the genetic determinants responsible for T2DM still remains unsolved. T2DM is not considered a genetic disease. Although recent papers demonstrate that the S20G mutation in the primary structure of hIAPP leads to an early variant of T2DM (18, 19), the incidence of this variant is very small compared to the number of patients affected by T2DM that do not show mutations in the hIAPP sequence. Moreover, studies focusing on the search for genetic factors involved in insulin resistance were inconclusive. For instance, defects on insulin receptors (INSR), correlated with insulin resistance, have been associated with T2DM, but only 3–4% of these patients show mutations of INSR genes (20). Other genes involved in triggering insulin resistance have been discovered: glucokinase regulatory protein (GKRP) (21), and insulin-like growth factor-I (IGF-I) (22). It has been reported that calpain-10 and later transcription factor 7-like two genes in affected individuals increase the risk to develop T2DM (23).

Recently, genetic factors involved in calcium dyshomeostasis have attracted much attention of scientists. It was noted that patients affected by familial hypercalciuric hypercalcemia, a genetic disease related to a reduction of calcium receptor gene expression, are much more prone to developing T2DM (24). This evidence supports a key role played by genetic factors regulating calcium dyshomeostasis in the development of T2DM. It is tempting to suggest that high plasma Ca^2+^ levels may activate pathogenic molecular mechanisms involving hIAPP-mediated membrane disruption, as already reported in literature (11, 25). It has been reported that there is an increase in the intracellular calcium ion concentration ([Ca^2+^]_i_) in diabetic subjects (26, 27). Notably, dysregulation of [Ca^2+^]_i_ in the cells of several tissues spontaneously occurs during aging as well as in obese people (28–30). Dyshomeostasis of intracellular Ca^2+^ can affect several membrane-related functions, such as domain organization, vesicular trafficking, and membrane adhesion/fusion (31, 32). A correlation between Ca^2+^ dyshomeostasis and hIAPP–membrane interaction (11, 25) has also been shown. In particular, it was observed that an increase of Ca^2+^ concentration facilitates hIAPP penetration into the phospholipid membrane to form pores (11).

## Weight Gain and T2DM: The Role of Free Fatty Acids

Several lifestyle factors, which characterize the most developed countries, affect the incidence of T2DM (33, 34). For example obesity or, more generally, weight gain, significantly increase the risk of developing the disease (35–37). Other risk factors are physical inactivity (35), habitual energy intake in relation to expenditure (35), composition of the diet (38, 39), and metabolic characteristics (35).

Insulin resistance, which is actually considered the starting point for the development of T2DM, is strictly correlated with weight gain (40, 41). From a molecular point of view, obesity leads to calcium dyshomeostasis (30), increased concentration of free fatty acids (FFA) (42), and cholesterol (43, 44). All of these effects have been shown to be involved in the molecular mechanisms leading to hIAPP-induced disruption of Langerhans’ β-cells (11, 25, 45, 46). Thus, these metabolic effects could all represent a common link between environmental factors and molecular mechanisms.

Plasma FFA concentration is usually elevated in obesity. It is worth noting that FFA may have a deep influence on Ca^2+^ homeostasis, which in turn triggers lipotoxic pathways leading to T2DM (47). Also, it has been shown that elevated FFA can cause insulin resistance and thus represents a common link between obesity, insulin resistance, and the development of T2DM. Elevation of plasma FFA levels in obesity is contributed by increased FFA release from enlarged adipose tissue, and reduced FFA clearance (48). Insulin resistance induced by high levels of FFA results in hyperinsulinemia and increased secretion of hIAPP. In turn, an increase in the local concentration of hIAPP mediates a more efficient interaction with the membrane resulting in pore formation and fibril elongation on the membrane surface (13). This conclusion is in agreement with previous studies demonstrating that high FFA concentrations enhance hIAPP fibrillogenicity (49).

To overcome the poor availability of isolated human islets, hIAPP-expressing transgenic mice were recently employed to investigate the role of high-fat diet on hIAPP amyloidosis. However, it is worth noting that amyloid growth in hIAPP-expressing transgenic mice and in human islets may significantly differ (50) since in human islets the amyloid aggregation starts intracellularly, while in transgenic mice amyloid growth is predominantly extracellular. It is important to point out that the diverse observations regarding whether hIAPP aggregation occurs intraceullarly or extraceullarly may likely depend on differing *in vivo* metabolic environments. It is tempting to speculate that differences in membrane lipid composition of rodents and human beings may play a major role in differentiating hIAPP behavior (51).

## Future Perspectives

T2DM is a pathology in which several factors are tightly interconnected. Mounting evidence points to a relationship between hIAPP amyloid, calcium dysregulation, membrane damage, and β-cell death. To date, inhibition of IAPP amyloid growth has been a matter of major interest. Overall, researcher efforts focusing on the design of molecules targeting hIAPP amyloid have been useful in providing different classes of anti-T2DM candidates (6, 52), however, to date, none of these compounds have been selected for clinical trials. Nowadays, the increasing knowledge of the mechanism of hIAPP toxicity moves the focus toward the development of molecules that can halt amyloid-mediated membrane damage. Since a two steps mechanism for membrane disruption mediated by hIAPP has been proposed, an efficient drug should inhibit both poration and fiber formation on membrane surface. However, there is still a lack of knowledge about the cross correlation of genetic, lifestyle and diet effects.

The use of model membranes allows researchers to study a simpler system than cells and to isolate single parameters affecting the process. An oversimplification in terms of lipid composition could lead, however, to misleading results. In order to fit *in vitro* and *in vivo* experiments with the process occurring in people affected by T2DM, future cell-free experiments should thus take into account the effects of genetic factors, lifestyle and diet. In particular, the effect of: (i) membrane composition, (ii) calcium ions, and (iii) FFA should be addressed by biophysical methods including Thioflavine T (ThT) fluorescence assays, dye-leakage, solid state NMR experiments, and large scale molecular simulations with the aim of providing a molecular description of the cascade of pathogenic events occurring upon hIAPP aggregation at water/lipid interfaces. Hopefully, these studies will help to effectively design and screen an increasingly large number of new compounds able to inhibit hIAPP aggregation also in the presence of lipid interfaces mimicking cell membranes and, in turn, to better manage IAPP toxicity in humans.

## Conflict of Interest Statement

The authors declare that the research was conducted in the absence of any commercial or financial relationships that could be construed as a potential conflict of interest.
